# Freshwater Biogeography and Limnological Evolution of the Tibetan Plateau - Insights from a Plateau-Wide Distributed Gastropod Taxon (*Radix* spp.)

**DOI:** 10.1371/journal.pone.0026307

**Published:** 2011-10-20

**Authors:** Parm Viktor von Oheimb, Christian Albrecht, Frank Riedel, Lina Du, Junxing Yang, David C. Aldridge, Ulrich Bößneck, Hucai Zhang, Thomas Wilke

**Affiliations:** 1 Department of Animal Ecology and Systematics, Justus Liebig University Giessen, Giessen, Germany; 2 Institute of Geological Sciences, Freie Universität Berlin, Berlin, Germany; 3 State Key Laboratory of Genetic Resources and Evolution, Chinese Academy of Sciences, Kunming, China; 4 Department of Zoology, Cambridge University, Cambridge, United Kingdom; 5 Natural History Museum of Erfurt, Erfurt, Germany; 6 Key Laboratory of Plateau Lake Ecology and Global Change, College of Tourism and Geography, Yunnan Normal University, Kunming, China; 7 State Key Laboratory of Lake Science and Environment, Chinese Academy of Sciences, Nanjing, China; State Natural History Museum, Germany

## Abstract

**Background:**

The Tibetan Plateau is not only the highest and largest plateau on earth; it is also home to numerous freshwater lakes potentially harbouring endemic faunal elements. As it remains largely unknown whether these lakes have continuously existed during the Last Glacial Maximum (LGM), questions arise as to whether taxa have been able to exist on the plateau since before the latest Pleistocene, from where and how often the plateau was colonized, and by which mechanisms organisms conquered remote high altitude lentic freshwater systems. In this study, species of the plateau-wide distributed freshwater gastropod genus *Radix* are used to answer these biogeographical questions.

**Methodology/Principal Findings:**

Based on a broad spatial sampling of *Radix* spp. on the Tibetan Plateau, and phylogenetic analyses of mtDNA sequence data, three probably endemic and one widespread major *Radix* clade could be identified on the plateau. Two of the endemic clades show a remarkably high genetic diversity, indicating a relatively great phylogenetic age. Phylogeographical analyses of individuals belonging to the most widely distributed clade indicate that intra-plateau distribution cannot be explained by drainage-related dispersal alone.

**Conclusions/Significance:**

Our study reveals that *Radix* spp. persisted throughout the LGM on the Tibetan Plateau. Therefore, we assume the continuous existence of suitable water bodies during that time. The extant *Radix* diversity on the plateau might have been caused by multiple colonization events combined with a relatively long intra-plateau evolution. At least one colonization event has a Palaearctic origin. In contrast to freshwater fishes, passive dispersal, probably by water birds, might be an important mechanism for conquering remote areas on the plateau. Patterns found in *Radix* spp. are shared with some terrestrial plateau taxa, indicating that *Radix* may be a suitable model taxon for inferring general patterns of biotic origin, dispersal and survival on the Tibetan Plateau.

## Introduction

The Tibetan Plateau located in central Asia is the highest and largest plateau on earth. The 2.5 million km^2^ large so-called “roof of the world” is surrounded by the Qilian and Altun Mountains in the north, by the Himalayas and Karakorum Mountains in the south and west, and by the Longmen Mountains in the east [1]. The uplift of the Tibetan Plateau probably started with the collision of Indian and Eurasian plates about 50 million years (Myr) ago [2]–[5]. Since Middle Miocene times (approx. 16–11 Myr ago), the elevation of the central and southern Tibetan Plateau has probably remained largely unchanged [6], [7], while in the east the growth continued until the Late Miocene [5]. Although the modern high elevation of the Tibetan Plateau of about 5,000 m a.s.l. [6] had been reached during the Miocene, regional differences in elevation histories are apparent. For south western Tibet, for example, a decrease in mean watershed elevation of 1,000 to 1,500 m since the Late Miocene is suggested [8].

The high elevation of the plateau is a primary reason for the existence of glaciers in these latitudes and ice-sheet formation certainly influenced the tectonic deformation regime at the regional level. Although there is evidence that the extent of glaciers on the Tibetan Plateau during the Last Glacial Maximum (LGM, approx. 20,000 ago) was larger than today, the interpretation of the spatio-temporal resolution remains controversial [9]–[12].

As the recent glaciers of the plateau hold the largest ice mass outside the Polar Regions [13], the “third pole” [14] is also of great importance for the freshwater systems of Asia. Many major rivers of South and East Asia originate on the Tibetan Plateau such as the Yellow, Yangtze, Mekong, Irrawaddy, Salween, Brahmaputra, and Indus rivers. Besides these lotic waters, about 1,600 lakes with sizes greater than 1 km^2^ and covering a total area of 50,900 km^2^ are scattered all over the plateau [15]. The exact origin and development of the plateau freshwater systems, especially lakes, however, is largely unknown. As the plateau lakes underwent massive lake level and thus environmental fluctuations at least during the late Quaternary [16], it is not clear how stable the freshwater systems on the Tibetan Plateau have been over time. In fact, it remains unknown whether and which lakes continuously existed on the plateau during the Pleistocene.

Although extensive geological studies are underway in order to obtain further insights into this topic, biogeographical analyses constitute an alternative approach for testing hypotheses about the evolution of freshwater systems. Patterns of freshwater biogeography are closely related to the history of associated freshwater systems as they are mainly products of historical processes [17]–[19]. If we assume that extant lentic freshwaters on the Tibetan Plateau have persisted since before the LGM, or even since before the Pleistocene, then the associated freshwater fauna should be relatively old and isolated. However, if such habitats did not exist continuously, extant taxa must have originated from post-LGM colonization of the Tibetan Plateau. In the latter case, the extant freshwater fauna on the Tibetan Plateau would have a young evolutionary history.

On the Tibetan Plateau, a number of studies have been carried out in recent years focusing on the evolutionary history and biogeography of animals (reviewed in [20]) as well as on plants [21], [22]. Although most of these studies targeted terrestrial organisms, some freshwater fish taxa of the area have been studied (e.g. [23]–[27]), contributing to a preliminary knowledge of regional freshwater biogeography and limnological evolution.

The plateau is usually assigned to the Palaearctic region [28], [29] with the Himalayan mountain ridge constituting the southern border to the Oriental region. Based on data from freshwater animals, especially of freshwater fish (Cyprinidae, Cobitidae, Sisoridae), Bănărescu [30], however, used a different classification scheme and considered High Asia (most of the Tibetan Plateau and surrounding mountain ranges) as a distinct subregion within the Sino-Indian region (South, East and Central Asia). Accordingly, the origin of the plateau ichthyo-fauna was supposed to be located in eastern and south-eastern Asia (i.e. in the Oriental region) [30].

However, a major limitation of plateau freshwater fishes as a biogeographical model is that, although widely present in large river systems, they are not known to be distributed plateau-wide. Unfortunately, the freshwater fauna of the Tibetan Plateau is quite species-poor (at least partly due to the high altitude [31]) and scarcely investigated, thus offering only few alternatives. Besides some Amphipoda and Ostracoda, it is assumed that two bivalve genera (*Pisidium*, *Musculium*), a genus of planorbid gastropods (*Gyraulus*) and particularly the lymnaeid gastropod genus *Radix* inhabit large parts of the plateau ([30], [32]–[34], P.V.v.O. personal observation). Populations of *Radix* spp. often occur in relatively high abundances in various water bodies including lakes, wetlands, and quiet parts of streams. Therefore, the genus *Radix* represents one of the very few useful model taxa for plateau-wide biogeographical investigations. The exact extent of the distribution range on the Tibetan Plateau as well as the biogeographical origin of the plateau lineages, however, is hitherto unknown for *Radix* spp.

An important factor for understanding the biogeography of plateau *Radix* spp. is the mechanism of these gastropods for conquering new habitats. For freshwater fishes from the Tibetan Plateau (Cyprinidae, Sisoridae) it was shown by using DNA sequence data that recent distribution patterns are strongly related to drainage areas, with drainage divides constituting insuperable barriers for dispersal. The resulting biogeographical patterns are thought to be linked to historical drainage dynamics [23], [24], and therefore strongly biased by the evolution of large river systems. As many freshwater gastropods are poor active dispersers with vector-mediated passive dispersal being the prevailing mode [35]–[37], they are probably less influenced by drainage histories. Given the wide distribution of *Radix* spp. on the Tibetan Plateau, this taxon also represents a good example for understanding the mechanisms taking faunal elements to the remote and disjunct freshwater systems of the plateau. Note that whereas dispersal of *Radix* spp. in large parts of the world is associated with human activities [38], this might not be the case on the Tibetan Plateau due to the sparse and relatively immobile human population.

Based on what we consider to be the most spatially comprehensive sampling of a freshwater taxon ever conducted on the Tibetan Plateau, we here use *Radix* spp. as a model for addressing three vexing questions concerning the biogeography and limnological evolution of Tibetan Plateau freshwater systems:

1. Did *Radix* spp. survive the LGM on the plateau, that is, did suitable freshwater habitats persist during the latest Pleistocene? The respective null hypothesis to be tested is:All recent lineages of *Radix* spp. originated from post-LGM colonization of the Tibetan Plateau.
2. What is the biogeographical origin of plateau *Radix* spp., that is, from where did the plateau receive its lymnaeid gastropod fauna? The respective null hypothesis to be tested is:Tibetan Plateau *Radix* spp. are exclusively of Oriental origin.
3. Are biogeographical patterns in *Radix* spp. mainly caused by limnological parameters such as drainage areas, that is, how did *Radix* spp. conquer the plateau's high altitude lentic freshwater systems? The respective null hypothesis to be tested is:The *Radix* distribution on the Tibetan Plateau can mainly be explained by drainage areas.

## Methods

### Sampling

Two general problems impede a comprehensive sampling of freshwater organisms on the Tibetan Plateau. First, many lakes in the central and northern part are endorheic and hyper saline [39], [40]. Thus, suitable habitats for freshwater organisms like *Radix* spp. are very scarce in this region. Second, large parts of the Tibetan Plateau are extremely remote and rugged, virtually uninhabited by humans, which makes them very difficult or impossible to access even by using a convoy of off-road vehicles. Given these problems, we performed extensive site-selection studies prior to the field work in order to optimize available resources and increase sampling success. Suitable water bodies for *Radix* spp. were identified based on literature information (e.g. [15], [39]) and analyses of satellite images (LANDSAT). We included in our sampling design through-flow lakes typically carrying freshwater as well as endorheic lakes fed by high amounts of melt water often exhibiting only low salinities. During the actual field work, only few of the pre-selected lakes were uninhabited by *Radix* spp. However, snails were also collected from some additional water bodies like streams, ponds and wetlands that were encountered during the structured field surveys.

**Figure 1:**
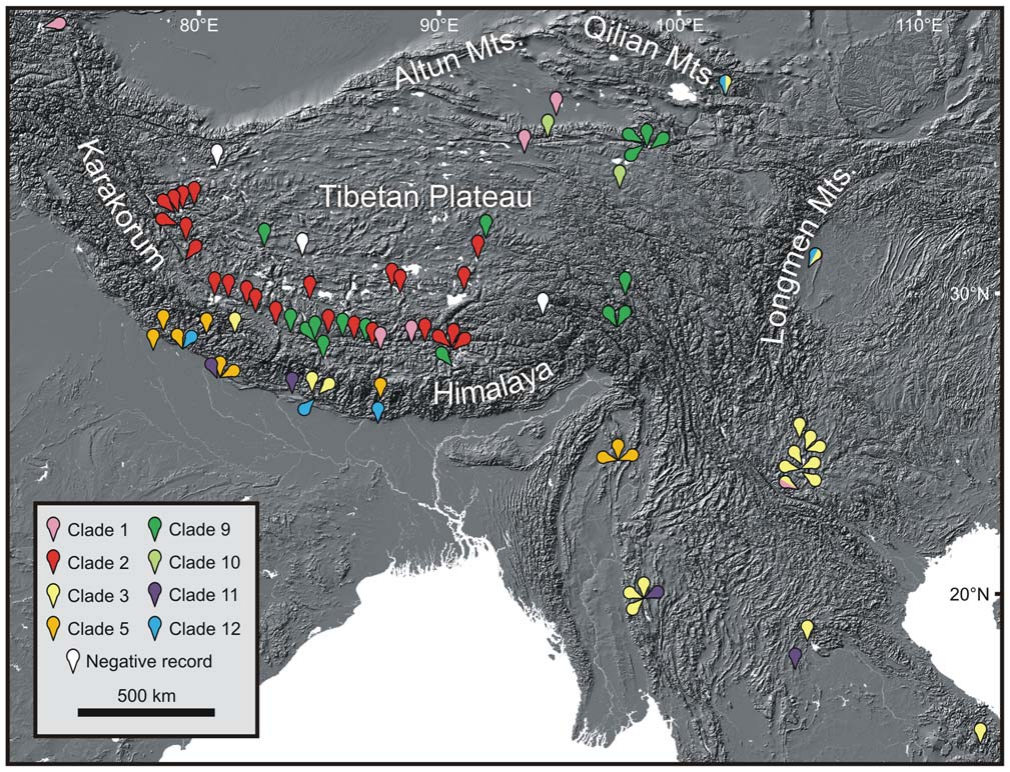
Sampling sites of *Radix* spp. in Asia and geographic distribution of major phylogenetic clades. The assignment to these clades is a result of the present study (see Figure 2 for details). Note that the locations of specimens belonging to clades 4 (Africa), 6 (Europe), 7 (northern and western Asia) and 8 (Europe) are not shown on the map. Only selected negative records of *Radix* spp. on the Tibetan Plateau are given.

During a total of 18 weeks of field work, specimens assigned to the genus *Radix* Montfort, 1810 (Pulmonata, Hygrophila, Lymnaeidae) were collected from 46 localities throughout most suitable parts of the Tibetan Plateau. Although our sampling is not equally dense on the whole plateau and far from complete, it appears to be largely representative given the unequal distribution of suitable habitats. In addition to the sampling in Tibet, *Radix* spp. were included from a total of 62 additional locations in various parts of Eurasia and in Africa. Special emphasis was placed on the adjacent areas of the Tibetan Plateau such as the southern slopes of the Himalayas and Southeast Asia (Figure 1, Table S1).

All specimens were preserved in 80% ethanol. Voucher materials (DNA and shell/tissue vouchers) are deposited at the Systematics and Biodiversity Collection of the University of Giessen (UGSB, see Table S1). All necessary permits for the field studies on the Tibetan Plateau were issued by the Chinese Academy of Sciences.

In the literature, various species names are assigned to *Radix* spp. from the Tibetan Plateau (primarily names of widespread Eurasian species, see e.g. [33]). The present study, however, shows that all plateau lineages of *Radix*, except one group closely related to *Radix auricularia* (here referred to as *R.* cf. *auricularia*), are endemic for the plateau in our dataset (see Result section). Available names of widespread species are thus not applicable to most Tibetan Plateau *Radix* spp. As a taxonomic revision is beyond the scope of the present paper and as the plateau lineages of *Radix* species will be formally described elsewhere, we here refer to the plateau *Radix* species as “*Radix* sp.” (see Table S1). For samples from outside the plateau, we use the available names only whenever unequivocal species assignment is possible.

### DNA isolation, PCR and sequencing

A total of 228 specimens was used for genetic analyses (see Table S1). DNA isolation [41] was done from foot tissue of individual gastropods. Then a 655 basepair (bp) fragment of the cytochrome *c* oxidase subunit I (COI) gene and a 429–440 bp fragment of the large ribosomal subunit (LSU rRNA) gene were amplified. COI primers were LCO1490 [42] and COR722b [43]. The latter primer is a slight modification of the Folmer et al. [42] primer HCO2198. For LSU rRNA, the primers 16Sar-L and 16Sbr-H [44] were used. Sequences (forward and reverse) were determined using the DNA sequencer Long Read IR 4200 (LI-COR, Lincoln, NE, USA) and the Thermo Sequenase Fluorescent Labeled Primer Cycle Sequencing kit (Amersham Pharmacia Biotech, Piscataway, NJ, USA). The COI sequences from eight additional *Radix* and the two outgroup taxa (*Planorbarius corneus* and *Physa fontinalis*) were taken from the literature [45].

### Sequence alignment

Alignment of outgroup and ingroup sequences was done individually for the COI and LSU rRNA genes. In the COI gene, the first bp behind the 3′ end of each primer were difficult to read. We therefore cut off the first and last bp of each sequence, leaving a 600 bp-long overlapping fragment. Alignment of the protein-coding COI sequences was carried out using Clustal W [46] implemented in BioEdit 7.0.9.0 [47]. The untruncated LSU rRNA sequences were aligned following the instructions for structural alignment by Kjer et al. [48]. The alignment was based on the LSU rRNA structure model of *Albinaria coerulea* [49] with ambiguous regions (especially loop regions) being aligned with PRANK [50]. For two regions (positions 42–69 and 328–379), no reliable alignment could be achieved. These regions were therefore excluded from subsequent phylogenetic analyses, leaving a 422 bp long LSU rRNA fragment, including gaps. The aligned dataset is available from the corresponding author upon request.

All sequences were deposited at GenBank (accession numbers JN794123-JN794514).

### Phylogenetic analyses

To assess the extant diversity of *Radix* spp. from the Tibetan Plateau (see Question 1) and to infer the biogeographical origin of individual lineages (see Question 2), phylogenetic analyses were conducted. First, the COI (codon positions 1/2 and 3 separately) and LSU rRNA datasets were individually tested for substitutional saturation using the entropy-based method of Xia et al. [51] as implemented in DAMBE 5.2.9 [52]. The test showed little saturation of both fragments under the assumption of a symmetrical tree.

Then, the COI (174 sequences) and LSU rRNA (228 sequences) data sets were combined according to the suggestions of Wiens [53]. Finally, identical sequences were removed from the combined dataset, leaving a total of 147 haplotypes (including the two outgroup taxa).

Phylogenetic relationships were inferred using Bayesian inference as implemented in the software package MrBayes v3.1.2 [54]. For the COI partition, the best fit HKY + I + G model [55] was used as suggested by the corrected Akaike information criterion (AICc) of jModelTest 0.1.1 [56], [57]. For the LSU rRNA partition, the 16B model [58] was applied to stem regions and the GTR + G model [59] to non-stem regions. The analyses were performed under two different tree models (ultrametric and non-ultrametric). For each model, two parallel runs were carried out with four chains (one cold, three heated). Analyses were terminated after achieving convergence of the parallel runs (split frequency standard deviations below the critical value of 0.01). This occurred after 5,243,000 generations (5,243 trees) and 5,032,000 generations (5,032 trees) for the ultrametric and non-ultrametric model, respectively. The mixing of the MCMC chains of the two runs was checked with TRACER v1.5.0 [60] and the burn-in defined as 10% of the sampled trees. The Bayes factor, that is the ratio of the marginal likelihoods of the two models [61], was then used to decide between the ultrametric and the non-ultrametric tree model. The marginal likelihoods were estimated using the harmonic mean of the post burn-in likelihood values of the MCMC samples calculated in MrBayes as ln = −8767.95 for the ultrametric tree model and ln = −8853.98 for the non-ultrametric tree model. The Bayes factor (2 ln) was estimated as being 172.06. According to Kass & Raftery [62] a Bayes factor above the value of 10 gives very strong evidence against the model with the lower likelihood value. Therefore, the phylogenetic trees inferred under the ultrametric tree model were combined to a consensus tree in TreeAnnotator v1.6.1 [60] and used for further investigations. Alternatively to Bayesian inference, the dataset was also analysed under the maximum-likelihood criterion using RAxML 7.0.3 [63], [64] with 1000 bootstrap replicates and the GTR + CAT approximation [64].

### Phylogeographical analyses

Phylogeographical analyses were utilized in order to infer patterns of intra-plateau *Radix* distribution (see Question 3). Network analyses were performed for the Tibetan Plateau clades 2, 9 and 10 and the Tibetan Plateau specimens belonging to clade 1 (see Result section) using TCS 1.21 [65]. Only those specimens were included for which both COI and LSU rRNA sequences were available. The LSU rRNA partitions of these reduced datasets were re-aligned according to the method described above, resulting in alignments with 432–437 positions (no highly variable parts had to be excluded in this data set containing only closely related taxa).

In order to get further insights into the mechanisms behind the distribution patterns of clade 2, the largest and most widely distributed clade in our dataset (Figure 1), the variation of genetic distances was partitioned based on different subsets of predictor variables using distance-based linear models in PERMANOVA+ for PRIMER [66]–[68]. First, average genetic distances between populations were calculated using MEGA 4 [69] under the K2P model and pairwise deletion of missing data. Then, the genetic distance matrix was related to the following subsets of environmental predictor variables: geographic location (latitude and longitude), drainage area (Brahmaputra, Indus or endorheic drainages) and elevation in a single model. Drainage area information was taken from the literature [70]. The various smaller drainages of the endorheic part of the Tibetan Plateau were summarized as “endorheic drainages”. Elevation data was inferred from a digital elevation model based on the SRTM (Shuttle Radar Topography Mission) dataset.

All *P*-values for the correlation of environmental predictors and genetic distances were obtained by permutation so that normality of errors is not assumed. A stepwise variable selection determined predictor subsets with the highest proportion of explained variation. The most parsimonious model was selected based on the AICc model selection criterion. Statistical significance of the model was tested using 10,000 permutations. As none of the correlations between individual predictor variables produced a Pearson correlation coefficient higher than 0.85, multicolinearity between predictor variables may be negligible [68].

## Results

**Figure 2:**
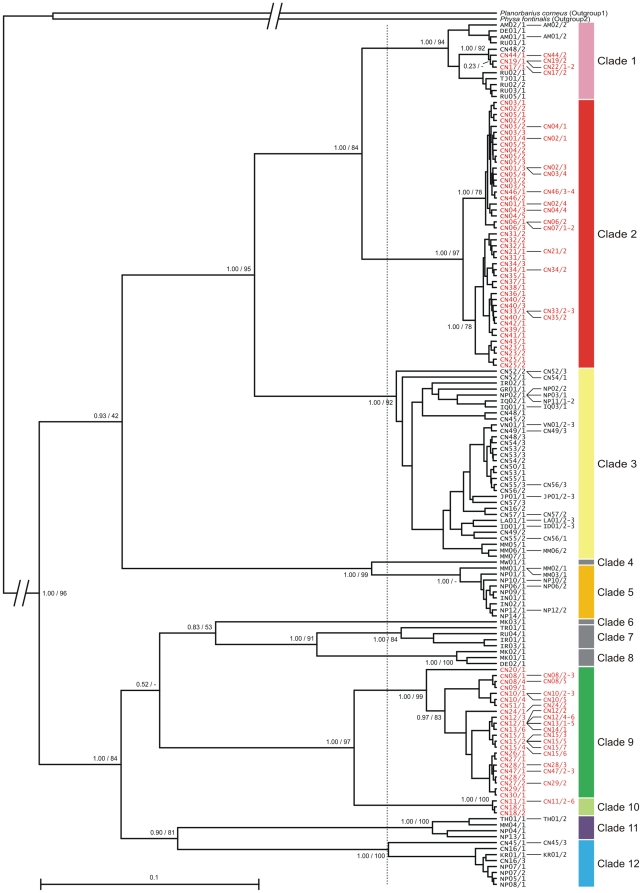
Consensus ultrametrical Bayesian inference tree of *Radix* spp. The tree was calculated under the ultrametric tree model based on the mitochondrial COI and LSU rRNA genes. Bayesian posterior probabilities (left) are provided for deeper nodes. Additionally, bootstrap values (right) of the alternative maximum likelihood analysis are given. The scale bar represents the substitutions per site according to the model of sequence evolution applied. Major clades (monophyletic groups younger than a node depth of 0.05 (grey line)) are labelled with bars. Specimens from the Tibetan Plateau are marked in red. For detailed specimen information see

The phylogenetic tree (Figure 2) shows that *Radix* spp. constitutes a highly diverse taxon, forming several “major” clades. In order to define these clades, we here use an objective, yet arbitrary criterion that relates to relative divergence time. All monophyletic groups being younger than a threshold value of a node depth of 0.05 substitutions per site in the ultrametric Bayesian tree (Figure 2) are regarded as “major clades”. The 12 resulting clades (named clade 1–12) are supported with Bayesian posterior probabilities (BPP) of 1.00, except for clades 4 and 6, each of which comprise only a single haplotype. Bootstrap support (BS) of the maximum likelihood analysis shows a similar picture (Figure 2).

Four different lineages of *Radix* were found on the Tibetan Plateau belonging to clades 1, 2, 9 and 10. Whereas in our dataset specimens of the latter three clades are restricted to the plateau, Tibetan Plateau specimens belonging to clade 1 (*Radix* cf. *auricularia*) cluster with lineages of the widespread species *Radix auricularia* (for species assignment see 1) and are most closely related to a specimen from southern China (CN48/2). The Tibetan Plateau specimens belonging to clade 1 were found on the southern plateau as well as on the north-eastern plateau (Figure 1). In contrast, clade 2 specimens are widely distributed over the western and central Tibetan Plateau. Clade 9 specimens occur on the central and eastern plateau and clade 10 specimens are restricted to the north-eastern plateau.

**Figure 3:**
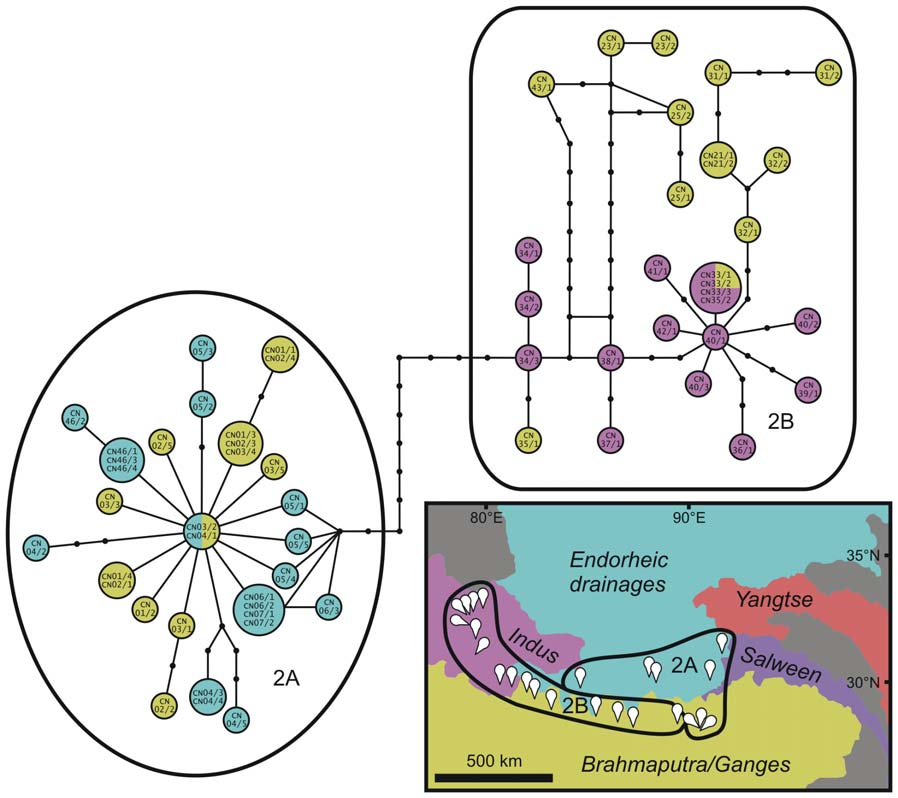
Statistical parsimony network of *Radix* clade 2 from the Tibetan Plateau. Colours refer to drainage areas where the respective specimens were collected. The geographic distribution of sub-groups (A, B) and drainage areas are shown on the map.

As for the phylogeography of the plateau clades, the network analysis of clade 2 revealed a single network with two genetically diverse sub-groups (2A and 2B in Figure 3). These sub-groups are also geographically distinct (i.e., distributions are not overlapping). Specimens from the Brahmaputra drainage fall into both sub-networks, whereas endorheic and Indus drainage specimens belong to different sub-networks. Drainage divides (between the Indus and Brahmaputra drainages and between the endorheic area and the Brahmaputra drainage) do not correspond to genetic breaks and identical haplotypes from different drainages were found in both sub-networks.

**Figure 4:**
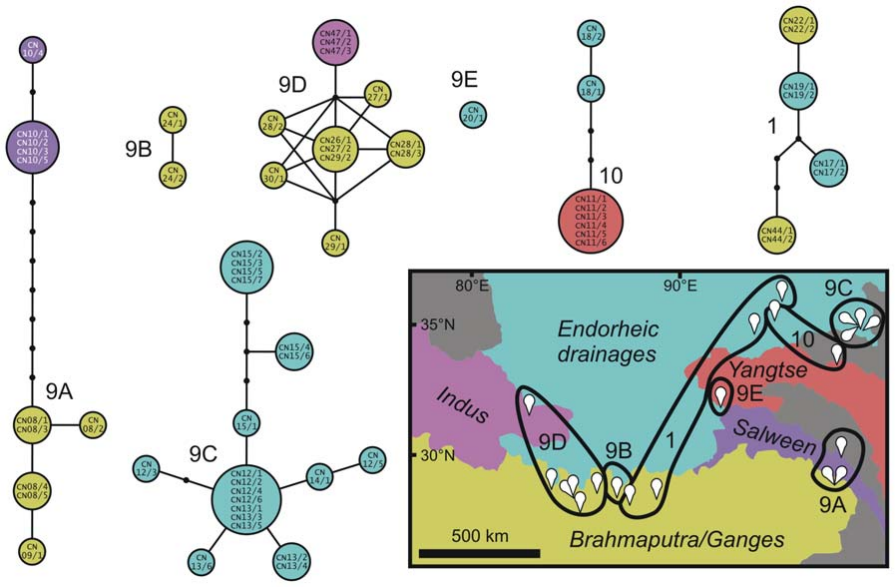
Statistical parsimony networks of *Radix* clades 1 (Tibetan subclade), 9 and 10 from the Tibetan Plateau. Colours refer to drainage areas where the respective specimens were collected. The geographic distribution of sub-groups of clade 9 (9A–9E) and drainage areas are shown on the map.

The Tibetan Plateau specimens belonging to clade 1 as well as clade 10 form single networks whereas clade 9 splits into five separate and geographically distinct networks (9A–9E) (Figure 4). Networks 1, 9A, 9D, and 10 each contain specimens originating from more than one drainage area. However, haplotypes are not shared across drainages.

Partitioning of genetic distance variation according to individual environmental predictor variables of populations belonging to clade 2 resulted in significant relations of geographic location (*R^2^* = 0.548; *P*<0.001) and drainage assignment (*R^2^* = 0.515; *P*<0.001) to genetic distance. Elevation alone showed no such relationship (*R^2^* = 0.092; *P* = 0.113). However, when analysing all three variables together, the AICc indicated geographic location and elevation as predictor subsets in the most parsimonious model. This model was able to explain 60.6% of the variation (*R^2^* = 0.606; *P*<0.001) in genetic distance.

## Discussion

### Did *Radix* spp. survive the LGM on the plateau, that is, did suitable freshwater habitats persist during the late Pleistocene?

If Tibetan Plateau *Radix* spp. originated from post-LGM colonization(s) of the plateau, as proposed in the respective null hypothesis, we would expect a very shallow phylogenetic structure with no or only few different mitochondrial haplotypes. Instead, we found two *Radix* lineages each with a remarkable genetic diversity on the plateau, indicating a relatively great phylogenetic age of these groups (see clades 2 and 9 in Figure 2 and the networks provided in Figures 3 and 4). Acknowledging that detailed molecular clock analyses are beyond the scope of this paper, the mean COI distances between the two most distantly related subclades within clade 2 and 9 can be calculated in MEGA 4 [69]. Applying the K2P model, resulting divergences are 3.1±0.6% for clade 2 and 6.5±0.9% for clade 9. Even assuming an extremely conservative COI divergence rate of 2.80%×Myr^−1^ (equalling a molecular clock rate of 1.40%×My^−1^) under the K2P model, the highest one noted by Wilke et al. [71], the observed divergences predate the LGM very clearly (>1 Myr).

The largest clade of exclusively Tibetan Plateau specimens found in our study, clade 2, is restricted to the western/central plateau in our dataset (Figure 1). The respective taxa are therefore likely the result of intra-plateau evolution. Clade 9 showed the highest intra-clade diversity of the plateau lineages. This might be the result of a pronounced diversification on the Tibetan Plateau, but multiple colonizations of the plateau by members of this clade cannot be ruled out. However, as clade 9 is the sister to clade 10, and both clades are endemic to the plateau in our dataset, multiple colonizations of clade 9 specimens appear to be unlikely. In fact, it is more likely that even the most recent common ancestor of clades 9 and 10 resided on the plateau and that these lineages therefore have an even longer intra-plateau evolutionary history. In contrast, the Tibetan Plateau *Radix* specimens belonging to clade 1 show little intra-plateau diversity and are closely related to a non-plateau specimen. As such they have probably a relatively short history of intra-plateau diversification.

Given these findings, the null hypothesis can be rejected and we assume that some but possibly not all lineages of *Radix* spp. persisted on the Tibetan Plateau during the LGM. Thus, suitable water bodies for *Radix* spp. had to be continuously present on the plateau during the LGM. It seems likely that at least some plateau lakes served as refugia for *Radix* spp. during the LGM as they are the deepest and therefore probably the most stable lentic habitats on the plateau. It can, however, not be excluded that other freshwater systems like springs, wetlands or streams also served as refugia. In other words, it remains unknown whether the lakes on the plateau share the same long history as *Radix* spp. or whether all or some of them functioned only as temporal stepping stones for the survival of populations.

### What is the biogeographical origin of plateau *Radix* spp., that is, from where did the plateau receive its lymnaeid gastropod fauna?

Given that Tibetan Plateau *Radix* spp. are not monophyletic (Figure 2), independent colonizations of the plateau have to be assumed. In order to infer the geographical origin of the respective founding specimens, knowledge about sister group relationships of plateau/non-plateau groups are useful.

The Tibetan Plateau specimens belonging to clade 1 cluster, together with a specimen from southern China, within *Radix auricularia* specimens from several parts of Eurasia. Because of this close relationship to Palaearctic taxa, the group can be considered as a Palaearctic element. The plateau endemic clade 2 is the sister group of clade 1 and may therefore also have a Palaearctic origin. The sister group of the remaining Tibetan Plateau clades, 9/10, consists of several specimens from Europe and western Asia (i.e. Palaearctic). This sister group relationship, however, is only poorly supported (BPP = 0.52). Moreover, given that specimens of clades 9 and 10 were found at the eastern part of the plateau and that our sampling from the adjacent mountain ranges in Yunnan and Sichuan is limited, an Oriental origin of these plateau groups cannot be excluded.

Nonetheless, based on the patterns observed, the Tibetan Plateau was most likely colonized by *Radix* spp. at least three times. At least one, possibly even all of these colonization events have a Palaearctic origin. Another interesting finding is the clear genetic break observed between southern and northern Himalayan *Radix* spp., indicating that the Himalayas present a sharp biogeographical barrier for *Radix* faunas.

Given the close relationship between some Tibetan Plateau and Palaearctic *Radix* taxa, we can reject the respective null hypothesis proposing an exclusively Oriental origin of the *Radix* fauna on the plateau. This finding stands in contrast to the patterns found for the plateau ichthyo-fauna, which is supposed to have derived from South and East Asian taxa exclusively (i.e. Oriental region) [30].

### Are biogeographical patterns in *Radix* spp. mainly caused by limnological parameters such as drainage areas, that is, how did *Radix* spp. conquer the plateau's high altitude lentic freshwater systems?

Our network analysis revealed a discordance of genetic groups and drainage areas for different major *Radix* clades. In fact, drainage divides do not correspond to genetic breaks and the respective null hypothesis, proposing that *Radix* distribution on the Tibetan Plateau can mainly be explained by drainage areas, can be rejected. This pattern contrasts the patterns found in freshwater fishes on the eastern plateau, where clades strongly correspond to drainage systems [23].

Moreover, our statistical modelling approach revealed only geographic location and elevation, but not drainage area, as significant predictors of the variation of genetic distances of clade 2 in the most parsimonious model. Based on these findings, active and/or passive dispersal along drainages is probably not the prevailing dispersal mechanism for Tibetan Plateau *Radix* spp. Instead, passive dispersal by vectors that can cross major drainage divides, probably water birds, might play an important role.

The ability of freshwater molluscs like *Radix* spp. to disperse passively among freshwater systems is much higher than those of freshwater fishes because of the potential to be transported by water birds in the plumage, attached to the feet or in the intestine [35], [36], [72]. These water birds are easily able to move among different drainages independent of drainage divides. More importantly they also can reach endorheic basins without hydrological connection to any main drainage, thus enabling *Radix* spp. to conquer diverse, remote and high altitude habitats on the Plateau.

### Intra-plateau biogeographical patterns

Our study shows that the intra-plateau biogeographical patterns found in Tibetan Plateau *Radix* spp. differ substantially from those found in freshwater fishes, probably due to major differences in habitat preferences and dispersal mechanisms.

As *Radix* individuals are obviously able to cross drainage divides (due to, e.g., passive dispersal via birds), respective biogeographical patterns may be more similar to terrestrial organisms than to fishes. Acknowledging that individual patterns can differ, two general biogeographical patterns have been found in terrestrial organisms from the Tibetan Plateau. On the one hand, relatively old groups with geographically distinct and genetically diverse lineages exist on the plateau. Examples for such groups are ground tits (*Pseudopodoces humilis*) [73], lizards (*Phrynocephalus vlangalii*) [74] and shrubs (*Hippophae tibetana*) [21]. In these taxa, past fragmentation might be caused by barriers and limited dispersal abilities. On the other hand, there are relatively young groups without a clear geographical structure and with low genetic diversity on the plateau, including perennial herbs (*Stellera chamaejasme*) [75] and junipers (*Juniperus przewalskii*) [76].

Interestingly, the same general patterns could also be observed in Tibetan Plateau *Radix* spp. Some taxa (see clades 2 and 9 in Figure 2) have a relatively high intra-plateau genetic diversity and constitute old and geographically distinct lineages. These groups thus clearly fit the former pattern, probably due to a relatively long history on the plateau and past fragmentation processes. In contrast, other taxa (see clade 1 in Figure 2) are phylogenetically young, lack a clear geographical structure on the plateau, and are characterized by a low genetic diversity. They therefore fit the latter pattern, indicating a recent colonization of the Plateau. Different lineages of plateau *Radix* spp. thus reflect different evolutionary histories covering different spatial and temporal scales. *Radix* spp. thus appears to have a high potential for inferring biogeographical processes on the Tibetan Plateau.

### Outlook

The present study focuses on one of the very few plateau-wide distributed freshwater taxa, the gastropod genus *Radix*. It provides new insights into the evolution of the freshwater systems of the Tibetan Plateau, closes biogeographical gaps and provides an important framework for further research on the geological and evolutionary history of one of the most remote places on earth.

Future studies could focus on the question of whether the plateau lakes *per se* or other water bodies, such as rivers and spring-supported wetlands, have served as refugia for *Radix* spp. during the Pleistocene. As biogeographical patterns found in plateau *Radix* spp. coincide with those found in other plateau taxa, they might be caused by historical processes on a regional (or global) scale rather than on a local scale. Thus, further research should combine biological, paleontological, geological and environmental studies in order to unravel the drivers of biotic evolution on the “roof of the world”.

## Supporting Information

Table S1 List of studied specimens including specimen code, taxon, locality and voucher details as well as GenBank accession numbers; voucher materials are deposited at the Systematics and Biodiversity Collection of the University of Giessen (UGSB).
(PDF)
