# Supplementary material for: Freshwater Biogeography and Limnological Evolution of the Tibetan Plateau - Insights from a Plateau-Wide Distributed Gastropod Taxon (Radix spp.)
Source: PLoS One. 2011 Oct 20;6(10):e26307. doi: 10.1371/journal.pone.0026307 (PMC3197626; doi:10.1371/journal.pone.0026307)
Supplement: Table S1 — List of studied specimens including specimen code, taxon, locality and voucher details as well as GenBank accession numbers; voucher materials are deposited at the Systematics and Biodiversity Collection of the University of Giessen (UGSB). (PDF) [file pone.0026307.s001.pdf]

Table S1. List of studied specimens including specimen code, taxon, locality and voucher details as well as GenBank accession numbers; voucher materials are deposited at the Systematics and Biodiversity Collection of the University of Giessen (UGSB).

Specimen code	Taxon	Locality	Latitude	Longitude	DNA voucher no.	Specimen voucher no.	COI GenBank accession no.	LSU rRNA GenBank accession no.
Outgroup1	*Planorbarius corneus* (Linnaeus, 1758)	Germany, Brandenburg, Altranft, dead river of River Alte Oder	52.76682°	14.09937°	F027	SMF 325460	EU818796	JN794123
Outgroup2	*Physa fontinalis* (Linnaeus, 1758)	Germany, Brandenburg, Obersdorf, Lake Vordersee	52.55120°	14.16850°	F024	UGSB 0316	AY282590	JN794124
AM01/1	*Radix auricularia* (Linnaeus, 1758)	Armenia, Kotayk Province, Hrazdan, inflow of reservoir	40.49962°	44.73923°	11097	UGSB 7318	JN794351	JN794125
AM01/2	*Radix auricularia* (Linnaeus, 1758)	Armenia, Kotayk Province, Hrazdan, inflow of reservoir	40.49962°	44.73923°	11098	UGSB 7319	JN794352	JN794126
AM02/1	*Radix auricularia* (Linnaeus, 1758)	Armenia, Gegharkunik Province, Tsakkar, river	40.18082°	45.22989°	11099	UGSB 7320	JN794353	JN794127
AM02/2	*Radix auricularia* (Linnaeus, 1758)	Armenia, Gegharkunik Province, Tsakkar, river	40.18082°	45.22989°	11100	UGSB 7321	JN794354	JN794128
CN01/1	*Radix* sp.	China, Tibet, Yamdrok Yumtso	29.09787°	90.37983°	10557	UGSB 7322	JN794355	JN794129
CN01/2	*Radix* sp.	China, Tibet, Yamdrok Yumtso	29.09787°	90.37983°	10558	UGSB 7323	JN794356	JN794130
CN01/3	*Radix* sp.	China, Tibet, Yamdrok Yumtso	29.09787°	90.37983°	11107	UGSB 7324	JN794357	JN794131
CN01/4	*Radix* sp.	China, Tibet, Yamdrok Yumtso	29.09787°	90.37983°	11108	UGSB 7325	JN794358	JN794132
CN02/1	*Radix* sp.	China, Tibet, Yamdrok Yumtso	29.09850°	90.37542°	10559	UGSB 7326	JN794359	JN794133
CN02/2	*Radix* sp.	China, Tibet, Yamdrok Yumtso	29.09850°	90.37542°	10560	UGSB 7327	JN794360	JN794134
CN02/3	*Radix* sp.	China, Tibet, Yamdrok Yumtso	29.09850°	90.37542°	10561	UGSB 7328	JN794361	JN794135
CN02/4	*Radix* sp.	China, Tibet, Yamdrok Yumtso	29.09850°	90.37542°	10562	UGSB 7329	JN794362	JN794136
CN02/5	*Radix* sp.	China, Tibet, Yamdrok Yumtso	29.09850°	90.37542°	11110	UGSB 7330	JN794363	JN794137
CN03/1	*Radix* sp.	China, Tibet, Yamdrok Yumtso	29.03494°	90.42122°	10050	UGSB 7331	JN794364	JN794138
CN03/2	*Radix* sp.	China, Tibet, Yamdrok Yumtso	29.03494°	90.42122°	10051	UGSB 7332	JN794365	JN794139
CN03/3	*Radix* sp.	China, Tibet, Yamdrok Yumtso	29.03494°	90.42122°	10052	UGSB 7333	JN794366	JN794140
CN03/4	*Radix* sp.	China, Tibet, Yamdrok Yumtso	29.03494°	90.42122°	10053	UGSB 7334	JN794367	JN794141
CN03/5	*Radix* sp.	China, Tibet, Yamdrok Yumtso	29.03494°	90.42122°	11111	UGSB 7335	JN794368	JN794142
CN04/1	*Radix* sp.	China, Tibet, Kyaring Tso	30.95592°	88.49245°	10054	UGSB 7336	JN794369	JN794143
CN04/2	*Radix* sp.	China, Tibet, Kyaring Tso	30.95592°	88.49245°	10055	UGSB 7337	JN794370	JN794144
CN04/3	*Radix* sp.	China, Tibet, Kyaring Tso	30.95592°	88.49245°	10056	UGSB 7338	JN794371	JN794145
CN04/4	*Radix* sp.	China, Tibet, Kyaring Tso	30.95592°	88.49245°	10057	UGSB 7339	JN794372	JN794146
CN04/5	*Radix* sp.	China, Tibet, Kyaring Tso	30.95592°	88.49245°	11112	UGSB 7340	JN794373	JN794147
CN05/1	*Radix* sp.	China, Tibet, Kyaring Tso	31.17519°	88.17454°	10058	UGSB 7341	JN794374	JN794148
CN05/2	*Radix* sp.	China, Tibet, Kyaring Tso	31.17519°	88.17454°	10059	UGSB 7342	JN794375	JN794149
CN05/3	*Radix* sp.	China, Tibet, Kyaring Tso	31.17519°	88.17454°	10060	UGSB 7343	JN794376	JN794150
CN05/4	*Radix* sp.	China, Tibet, Kyaring Tso	31.17519°	88.17454°	10061	UGSB 7344	JN794377	JN794151
CN05/5	*Radix* sp.	China, Tibet, Kyaring Tso	31.17519°	88.17454°	11113	UGSB 7345	JN794378	JN794152
CN06/1	*Radix* sp.	China, Tibet, Tso Nak	32.02789°	91.53130°	10563	UGSB 7346	JN794379	JN794153
CN06/2	*Radix* sp.	China, Tibet, Tso Nak	32.02789°	91.53130°	10564	UGSB 7347	JN794380	JN794154
CN06/3	*Radix* sp.	China, Tibet, Tso Nak	32.02789°	91.53130°	10934	UGSB 7348	JN794381	JN794155
CN07/1	*Radix* sp.	China, Tibet, small lake north of Nam Tso Chukmo	30.99924°	90.94743°	10565	UGSB 7349	JN794382	JN794156
CN07/2	*Radix* sp.	China, Tibet, small lake north of Nam Tso Chukmo	30.99924°	90.94743°	10566	UGSB 7350	JN794383	JN794157
CN08/1	*Radix* sp.	China, Tibet, small ponds near Rawok Tso	29.48598°	96.66630°	10062	UGSB 7351	JN794384	JN794158
CN08/2	*Radix* sp.	China, Tibet, small ponds near Rawok Tso	29.48598°	96.66630°	10063	UGSB 7352	JN794385	JN794159
CN08/3	*Radix* sp.	China, Tibet, small ponds near Rawok Tso	29.48598°	96.66630°	10064	UGSB 7353	JN794386	JN794160
CN08/4	*Radix* sp.	China, Tibet, small ponds near Rawok Tso	29.48598°	96.66630°	10568	UGSB 7354	JN794387	JN794161
CN08/5	*Radix* sp.	China, Tibet, small ponds near Rawok Tso	29.48598°	96.66630°	10569	UGSB 7355	JN794388	JN794162
CN09/1	*Radix* sp.	China, Tibet, Rawok Tso	29.48598°	96.66630°	10570	UGSB 7356	JN794389	JN794163

CN10/1	*Radix* sp.	China, Tibet, lake near Bangda Airport	30.49675°	97.07836°	10571	UGSB 7357	JN794390	JN794164
CN10/2	*Radix* sp.	China, Tibet, lake near Bangda Airport	30.49675°	97.07836°	10573	UGSB 7358	JN794391	JN794165
CN10/3	*Radix* sp.	China, Tibet, lake near Bangda Airport	30.49675°	97.07836°	10574	UGSB 7359	JN794392	JN794166
CN10/4	*Radix* sp.	China, Tibet, lake near Bangda Airport	30.49675°	97.07836°	10575	UGSB 7360	JN794393	JN794167
CN10/5	*Radix* sp.	China, Tibet, lake near Bangda Airport	30.49675°	97.07836°	10576	UGSB 7361	JN794394	JN794168
CN11/1	*Radix* sp.	China, Qinghai, pond at lake near Chumatang	34.00598°	97.26157°	10065	UGSB 7362	JN794395	JN794169
CN11/2	*Radix* sp.	China, Qinghai, pond at lake near Chumatang	34.00598°	97.26157°	10066	UGSB 7363	JN794396	JN794170
CN11/3	*Radix* sp.	China, Qinghai, pond at lake near Chumatang	34.00598°	97.26157°	10067	UGSB 7364	JN794397	JN794171
CN11/4	*Radix* sp.	China, Qinghai, pond at lake near Chumatang	34.00598°	97.26157°	10577	UGSB 7365	JN794398	JN794172
CN11/5	*Radix* sp.	China, Qinghai, pond at lake near Chumatang	34.00598°	97.26157°	10578	UGSB 7366	JN794399	JN794173
CN11/6	*Radix* sp.	China, Qinghai, pond at lake near Chumatang	34.00598°	97.26157°	10579	UGSB 7367	JN794400	JN794174
CN12/1	*Radix* sp.	China, Qinghai, ponds and floodplains next to Dongi Tsona	35.37019°	98.49866°	10068	UGSB 7368	JN794401	JN794175
CN12/2	*Radix* sp.	China, Qinghai, ponds and floodplains next to Dongi Tsona	35.37019°	98.49866°	10069	UGSB 7369	JN794402	JN794176
CN12/3	*Radix* sp.	China, Qinghai, ponds and floodplains next to Dongi Tsona	35.37019°	98.49866°	10070	UGSB 7370	JN794403	JN794177
CN12/4	*Radix* sp.	China, Qinghai, ponds and floodplains next to Dongi Tsona	35.37019°	98.49866°	10935	UGSB 7371	JN794404	JN794178
CN12/5	*Radix* sp.	China, Qinghai, ponds and floodplains next to Dongi Tsona	35.37019°	98.49866°	10936	UGSB 7372	JN794405	JN794179
CN12/6	*Radix* sp.	China, Qinghai, ponds and floodplains next to Dongi Tsona	35.37019°	98.49866°	10937	UGSB 7373	JN794406	JN794180
CN13/1	*Radix* sp.	China, Qinghai, Dongi Tsona	35.38323°	98.46711°	10938	UGSB 7374	JN794407	JN794181
CN13/2	*Radix* sp.	China, Qinghai, Dongi Tsona	35.38323°	98.46711°	10939	UGSB 7375	JN794408	JN794182
CN13/3	*Radix* sp.	China, Qinghai, Dongi Tsona	35.38323°	98.46711°	10940	UGSB 7376	JN794409	JN794183
CN13/4	*Radix* sp.	China, Qinghai, Dongi Tsona	35.38323°	98.46711°	10941	UGSB 7377	JN794410	JN794184
CN13/5	*Radix* sp.	China, Qinghai, Dongi Tsona	35.38323°	98.46711°	10942	UGSB 7378	JN794411	JN794185
CN13/6	*Radix* sp.	China, Qinghai, Dongi Tsona	35.38323°	98.46711°	10943	UGSB 7379	JN794412	JN794186
CN14/1	*Radix* sp.	China, Qinghai, Dongi Tsona	35.25400°	98.50352°	10944	UGSB 7380	JN794413	JN794187
CN15/1	*Radix* sp.	China, Qinghai, small lake south of Dongi Tsona	35.19938°	98.60763°	10071	UGSB 7381	JN794414	JN794188
CN15/2	*Radix* sp.	China, Qinghai, small lake south of Dongi Tsona	35.19938°	98.60763°	10072	UGSB 7382	JN794415	JN794189
CN15/3	*Radix* sp.	China, Qinghai, small lake south of Dongi Tsona	35.19938°	98.60763°	10073	UGSB 7383	JN794416	JN794190
CN15/4	*Radix* sp.	China, Qinghai, small lake south of Dongi Tsona	35.19938°	98.60763°	10945	UGSB 7384	JN794417	JN794191
CN15/5	*Radix* sp.	China, Qinghai, small lake south of Dongi Tsona	35.19938°	98.60763°	10946	UGSB 7385	JN794418	JN794192
CN15/6	*Radix* sp.	China, Qinghai, small lake south of Dongi Tsona	35.19938°	98.60763°	10947	UGSB 7386	JN794419	JN794193
CN15/7	*Radix* sp.	China, Qinghai, small lake south of Dongi Tsona	35.19938°	98.60763°	10948	UGSB 7387	JN794420	JN794194
CN16/1	*Radix* sp.	China, Qinghai, Huangshui River	36.56303°	101.89902°	12033	UGSB 7388	JN794421	JN794195
CN16/2	*Radix* sp.	China, Qinghai, Huangshui River	36.56303°	101.89902°	12048	UGSB 7389		JN794196
CN16/3	*Radix* sp.	China, Qinghai, Huangshui River	36.56303°	101.89902°	12420	UGSB 7390	JN794422	JN794197
CN17/1	*Radix* cf. *auricularia* (Linnaeus, 1758)	China, Qinghai, pond north of Gormo	36.59840°	95.00298°	12034	UGSB 7391	JN794423	JN794198
CN17/2	*Radix* cf. *auricularia* (Linnaeus, 1758)	China, Qinghai, pond north of Gormo	36.59840°	95.00298°	12421	UGSB 7392	JN794424	JN794199
CN18/1	*Radix* sp.	China, Qinghai, pond north of Naij Tal	35.88329°	94.57995°	12035	UGSB 7393	JN794425	JN794200
CN18/2	*Radix* sp.	China, Qinghai, pond north of Naij Tal	35.88329°	94.57995°	12050	UGSB 7394	JN794426	JN794201
CN19/1	*Radix* cf. *auricularia* (Linnaeus, 1758)	China, Qinghai, small river west of Budongquan	35.43374°	93.60115°	12036	UGSB 7395	JN794427	JN794202
CN19/2	*Radix* cf. *auricularia* (Linnaeus, 1758)	China, Qinghai, small river west of Budongquan	35.43374°	93.60115°	12051	UGSB 7396	JN794428	JN794203
CN20/1	*Radix* sp.	China, Tibet, small river with pond north of Tso Nak	32.69793°	91.87753°	12037	UGSB 7397	JN794429	JN794204
CN21/1	*Radix* sp.	China, Tibet, small lake between Lhasa and Shigatse	29.32508°	89.40617°	12038	UGSB 7398	JN794430	JN794205
CN21/2	*Radix* sp.	China, Tibet, small lake between Lhasa and Shigatse	29.32508°	89.40617°	12053	UGSB 7399	JN794431	JN794206
CN22/1	*Radix* cf. *auricularia* (Linnaeus, 1758)	China, Tibet, pond east of Chushar	29.06212°	87.71676°	12039	UGSB 7400	JN794432	JN794207
CN22/2	*Radix* cf. *auricularia* (Linnaeus, 1758)	China, Tibet, pond east of Chushar	29.06212°	87.71676°	12054	UGSB 7401	JN794433	JN794208
CN23/1	*Radix* sp.	China, Tibet, Lang Tso	29.21256°	87.42069°	12318	UGSB 7402	JN794434	JN794209
CN23/2	*Radix* sp.	China, Tibet, Lang Tso	29.21256°	87.42069°	12319	UGSB 7403	JN794435	JN794210
CN24/1	*Radix* sp.	China, Tibet, small inflow of Ngamring Tso	29.34203°	87.16489°	12040	UGSB 7404	JN794436	JN794211
CN24/2	*Radix* sp.	China, Tibet, small inflow of Ngamring Tso	29.34203°	87.16489°	12055	UGSB 7405	JN794437	JN794212

CN25/1	*Radix* sp.	China, Tibet, pond near Sangsang	29.41796°	86.72390°	12320	UGSB 7406	JN794438	JN794213
CN25/2	*Radix* sp.	China, Tibet, pond near Sangsang	29.41796°	86.72390°	12321	UGSB 7407	JN794439	JN794214
CN26/1	*Radix* sp.	China, Tibet, small lake east of Charang Tso	29.51514°	86.28578°	12041	UGSB 7408	JN794440	JN794215
CN27/1	*Radix* sp.	China, Tibet, pond at Brahmaputra near Kyakyaru	29.32303°	85.28699°	12322	UGSB 7409	JN794441	JN794216
CN27/2	*Radix* sp.	China, Tibet, pond at Brahmaputra near Kyakyaru	29.32303°	85.28699°	12323	UGSB 7410	JN794442	JN794217
CN28/1	*Radix* sp.	China, Tibet, inflow of Palku Tso	28.77180°	85.56133°	12042	UGSB 7411	JN794443	JN794218
CN28/2	*Radix* sp.	China, Tibet, inflow of Palku Tso	28.77180°	85.56133°	12057	UGSB 7412	JN794444	JN794219
CN28/3	*Radix* sp.	China, Tibet, inflow of Palku Tso	28.77180°	85.56133°	12665	UGSB 7413	JN794445	JN794220
CN29/1	*Radix* sp.	China, Tibet, wetlands north of Kyakyaru	29.40823°	85.24297°	12058	UGSB 7414	JN794446	JN794221
CN29/2	*Radix* sp.	China, Tibet, wetlands north of Kyakyaru	29.40823°	85.24297°	12324	UGSB 7415	JN794447	JN794222
CN30/1	*Radix* sp.	China, Tibet, pond near Drongpa Tradun	29.64008°	84.30929°	12059	UGSB 7416	JN794448	JN794223
CN31/1	*Radix* sp.	China, Tibet, wetlands west of Drongpa Tradun	29.87067°	83.73763°	12060	UGSB 7417	JN794449	JN794224
CN31/2	*Radix* sp.	China, Tibet, wetlands west of Drongpa Tradun	29.87067°	83.73763°	12667	UGSB 7418	JN794450	JN794225
CN32/1	*Radix* sp.	China, Tibet, small stream west of Baryang	30.26702°	82.95391°	12325	UGSB 7419	JN794451	JN794226
CN32/2	*Radix* sp.	China, Tibet, small stream west of Baryang	30.26702°	82.95391°	12326	UGSB 7420	JN794452	JN794227
CN33/1	*Radix* sp.	China, Tibet, small stream east of Lake Manasarovar	30.70560°	81.90172°	12061	UGSB 7421	JN794453	JN794228
CN33/2	*Radix* sp.	China, Tibet, small stream east of Lake Manasarovar	30.70560°	81.90172°	12668	UGSB 7422	JN794454	JN794229
CN33/3	*Radix* sp.	China, Tibet, small stream east of Lake Manasarovar	30.70560°	81.90172°	12669	UGSB 7423	JN794455	JN794230
CN34/1	*Radix* sp.	China, Tibet, Lake Manasarovar	30.76365°	81.37367°	12062	UGSB 7424	JN794456	JN794231
CN34/2	*Radix* sp.	China, Tibet, Lake Manasarovar	30.76365°	81.37367°	12670	UGSB 7425	JN794457	JN794232
CN34/3	*Radix* sp.	China, Tibet, Lake Manasarovar	30.76365°	81.37367°	12671	UGSB 7426	JN794458	JN794233
CN35/1	*Radix* sp.	China, Tibet, small stream east of Gung-gyu Tso	30.51992°	82.60483°	12063	UGSB 7427	JN794459	JN794234
CN35/2	*Radix* sp.	China, Tibet, small stream east of Gung-gyu Tso	30.51992°	82.60483°	12672	UGSB 7428	JN794460	JN794235
CN36/1	*Radix* sp.	China, Tibet, small lake south of Senge Tsangpo	31.88762°	80.16509°	12064	UGSB 7429	JN794461	JN794236
CN37/1	*Radix* sp.	China, Tibet, Indus River near Senge Tsangpo	32.50931°	80.14338°	12065	UGSB 7430	JN794462	JN794237
CN38/1	*Radix* sp.	China, Tibet, stream north of Senge Tsangpo	33.03478°	79.81388°	12066	UGSB 7431	JN794463	JN794238
CN39/1	*Radix* sp.	China, Tibet, pond near Pangong Tso	33.41844°	79.64039°	12067	UGSB 7432	JN794464	JN794239
CN40/1	*Radix* sp.	China, Tibet, Pangong Tso	33.44151°	79.78342°	12043	UGSB 7433	JN794465	JN794240
CN40/2	*Radix* sp.	China, Tibet, Pangong Tso	33.44151°	79.78342°	12044	UGSB 7434	JN794466	JN794241
CN40/3	*Radix* sp.	China, Tibet, Pangong Tso	33.44151°	79.78342°	12045	UGSB 7435	JN794467	JN794242
CN41/1	*Radix* sp.	China, Tibet, Pangong Tso	33.55384°	79.92984°	12068	UGSB 7436	JN794468	JN794243
CN42/1	*Radix* sp.	China, Tibet, small stream in Domar	33.71328°	80.37660°	12069	UGSB 7437	JN794469	JN794244
CN43/1	*Radix* sp.	China, Tibet, Yadang Tso	29.63267°	85.74019°	12071	UGSB 7438	JN794470	JN794245
CN44/1	*Radix* cf. *auricularia* (Linnaeus, 1758)	China, Tibet, pond in Shigatse	29.27210°	88.89421°	12673	UGSB 7439	JN794471	JN794246
CN44/2	*Radix* cf. *auricularia* (Linnaeus, 1758)	China, Tibet, pond in Shigatse	29.27210°	88.89421°	12674	UGSB 7440	JN794472	JN794247
CN45/1	*Radix* sp.	China, Sichuan, Chengdu, small artificial ditch	30.66032°	104.05583°	14810	UGSB 7441	JN794473	JN794248
CN45/2	*Radix* sp.	China, Sichuan, Chengdu, small artificial ditch	30.66032°	104.05583°	15036	UGSB 7442		JN794249
CN45/3	*Radix* sp.	China, Sichuan, Chengdu, small artificial ditch	30.66032°	104.05583°	15037	UGSB 7443	JN794474	JN794250
CN46/1	*Radix* sp.	China, Tibet, Kyering Tso	30.76610°	85.01230°	15122	UGSB 7444	JN794475	JN794251
CN46/2	*Radix* sp.	China, Tibet, Kyering Tso	30.76610°	85.01230°	15123	UGSB 7445	JN794476	JN794252
CN46/3	*Radix* sp.	China, Tibet, Kyering Tso	30.76610°	85.01230°	15124	UGSB 7446	JN794477	JN794253
CN46/4	*Radix* sp.	China, Tibet, Kyering Tso	30.76610°	85.01230°	15125	UGSB 7447	JN794478	JN794254
CN47/1	*Radix* sp.	China, Tibet, wetlands south of Tarab Tso	32.44102°	83.20563°	15126	UGSB 7448	JN794479	JN794255
CN47/2	*Radix* sp.	China, Tibet, wetlands south of Tarab Tso	32.44102°	83.20563°	15128	UGSB 7449	JN794480	JN794256
CN47/3	*Radix* sp.	China, Tibet, wetlands south of Tarab Tso	32.44102°	83.20563°	15129	UGSB 7450	JN794481	JN794257
CN48/1	*Radix* sp.	China, Yunnan, Lake Yilong	23.65618°	102.62479°	12024	UGSB 7451		JN794258
CN48/2	*Radix* sp.	China, Yunnan, Lake Yilong	23.65618°	102.62479°	12025	UGSB 7452		JN794259
CN48/3	*Radix* sp.	China, Yunnan, Lake Yilong	23.65618°	102.62479°	12026	UGSB 7453		JN794260
CN49/1	*Radix* sp.	China, Yunnan, Daying dragon spring	25.27322°	102.85972°	12027	UGSB 7454		JN794261

CN49/2	*Radix* sp.	China, Yunnan, Daying dragon spring	25.27322°	102.85972°	12028	UGSB 7455		JN794262
CN49/3	*Radix* sp.	China, Yunnan, Daying dragon spring	25.27322°	102.85972°	12029	UGSB 7456		JN794263
CN50/1	*Radix* sp.	China, Yunnan, Lake Xingyun	24.30998°	102.76592°	12030	UGSB 7457		JN794264
CN51/1	*Radix* sp.	China, Tibet, Phuma Yumtso	28.49536°	90.42641°	12422	UGSB 7458		JN794265
CN52/1	*Radix* sp.	China, Yunnan, Lake Fuxian	24.43796°	102.85104°	12303	UGSB 7459		JN794266
CN52/2	*Radix* sp.	China, Yunnan, Lake Fuxian	24.43796°	102.85104°	12304	UGSB 7460		JN794267
CN52/3	*Radix* sp.	China, Yunnan, Lake Fuxian	24.43796°	102.85104°	12305	UGSB 7461		JN794268
CN53/1	*Radix* sp.	China, Yunnan, Lake Xingyun	24.38078°	102.80719°	12306	UGSB 7462	JN794482	JN794269
CN53/2	*Radix* sp.	China, Yunnan, Lake Xingyun	24.38078°	102.80719°	12307	UGSB 7463	JN794483	JN794270
CN53/3	*Radix* sp.	China, Yunnan, Lake Xingyun	24.38078°	102.80719°	12308	UGSB 7464	JN794484	JN794271
CN54/1	*Radix* sp.	China, Yunnan, Lake Qilu	24.19123°	102.81068°	12309	UGSB 7465		JN794272
CN54/2	*Radix* sp.	China, Yunnan, Lake Qilu	24.19123°	102.81068°	12310	UGSB 7466	JN794485	JN794273
CN54/3	*Radix* sp.	China, Yunnan, Lake Qilu	24.19123°	102.81068°	12311	UGSB 7467	JN794486	JN794274
CN55/1	*Radix* sp.	China, Yunnan, Lake Yangzong	24.86115°	102.99083°	12312	UGSB 7468	JN794487	JN794275
CN55/2	*Radix* sp.	China, Yunnan, Lake Yangzong	24.86115°	102.99083°	12313	UGSB 7469		JN794276
CN55/3	*Radix* sp.	China, Yunnan, Lake Yangzong	24.86115°	102.99083°	12314	UGSB 7470	JN794488	JN794277
CN56/1	*Radix* sp.	China, Yunnan, Lake Yangzong	24.87302°	103.00794°	12315	UGSB 7471		JN794278
CN56/2	*Radix* sp.	China, Yunnan, Lake Yangzong	24.87302°	103.00794°	12316	UGSB 7472	JN794489	JN794279
CN56/3	*Radix* sp.	China, Yunnan, Lake Yangzong	24.87302°	103.00794°	12317	UGSB 7473	JN794490	JN794280
CN57/1	*Radix* sp.	China, Beijing, Huanghuacheng	40.40722°	116.31833°	14811	UGSB 7474		JN794281
CN57/2	*Radix* sp.	China, Beijing, Huanghuacheng	40.40722°	116.31833°	14812	UGSB 7475		JN794282
CN57/3	*Radix* sp.	China, Beijing, Huanghuacheng	40.40722°	116.31833°	15038	UGSB 7476		JN794283
DE01/1	*Radix auricularia* (Linnaeus, 1758)	Germany, Thuringia, Saalfeld-Rudolstadt district, tempory pool N of Birkigt	50.67280°	11.48558°	F119	UGSB 0300	EU818800	JN794284
DE02/1	*Radix ampla* (Hartmann, 1821)	Germany, Brandenburg, Gülpe, inflow of Lake Gülpe	52.75343°	12.31504°	6391	UGSB 0301	EU818836	JN794285
GR01/1	*Radix* sp. 2 sensu Albrecht et al. [45]	Greece, Aetoloakarnania, Lake Trichonis, NW shore	38.58893°	21.46703°	5433	UGSB 0308	EU818823	JN794286
ID01/1	*Radix* sp.	Indonesia, South Sulawesi, river	-4.62763°	119.62700°	9382	UGSB 7477		JN794287
ID01/2	*Radix* sp.	Indonesia, South Sulawesi, river	-4.62763°	119.62700°	9383	UGSB 7478		JN794288
ID01/3	*Radix* sp.	Indonesia, South Sulawesi, river	-4.62763°	119.62700°	9384	UGSB 7479		JN794289
IN01/1	*Radix* sp.	India, Uttarakhand, Kosi River west of Almora	29.49667°	79.50222°	11246	UGSB 7480		JN794290
IN02/1	*Radix* sp.	India, Uttar Pradesh, Kosi River north of Rampur	28.84528°	79.16500°	11248	UGSB 7481	JN794491	JN794291
IQ01/1	*Radix* sp.	Iraq, Maysan Governorate, Hammar Marshes	31.20037°	46.99545°	11146	UGSB 7482		JN794292
IQ02/1	*Radix* sp.	Iraq, Maysan Governorate, Hammar Marshes	31.20037°	46.99545°	11147	UGSB 7483		JN794293
IQ03/1	*Radix* sp.	Iraq, Basra Governorate, Garmat Ali River	30.59494°	47.69812°	11149	UGSB 7484		JN794294
IR01/1	*Radix* sp.	Iran, Lorestan Province, stream near Mode Abad village	33.58238°	49.61093°	11155	UGSB 7485		JN794295
IR02/1	*Radix* sp.	Iran, North Khorasan Province,spring near Shirvan	37.33333°	57.66667°	11156	UGSB 7486		JN794296
IR03/1	*Radix* sp.	Iran, Markazi Province, spring in Emamzadeh Varcheh village	33.81667°	49.91667°	11159	UGSB 7487		JN794297
JP01/1	*Radix* sp.	Japan, Kyushu, Saga Prefecture, Kiyama, river	33.42330°	130.53200°	9376	UGSB 7488		JN794298
JP01/2	*Radix* sp.	Japan, Kyushu, Saga Prefecture, Kiyama, river	33.42330°	130.53200°	9377	UGSB 7489		JN794299
JP01/3	*Radix* sp.	Japan, Kyushu, Saga Prefecture, Kiyama, river	33.42330°	130.53200°	9378	UGSB 7490		JN794300
KR01/1	*Radix* sp.	South Korea, Gangwon-do, river south of Gangneung	37.57300°	128.85200°	9385	UGSB 7491	JN794492	JN794301
KR01/2	*Radix* sp.	South Korea, Gangwon-do, river south of Gangneung	37.57300°	128.85200°	9386	UGSB 7492		JN794302
LA01/1	*Radix* sp.	Laos, Vientiane Province, Xong River	18.75350°	102.40100°	9373	UGSB 7493		JN794303
LA01/2	*Radix* sp.	Laos, Vientiane Province, Xong River	18.75350°	102.40100°	9374	UGSB 7494		JN794304
LA01/3	*Radix* sp.	Laos, Vientiane Province, Xong River	18.75350°	102.40100°	9375	UGSB 7495		JN794305
MK01/1	*Radix* sp. 1 sensu Albrecht et al. [45]	Macedonia, Sv. Naum, at SE shore of Lake Ohrid	40.91275°	20.74502°	5481	UGSB 0305	EU818825	JN794306
MK02/1	*Radix relicta* (Polinski, 1929)	Macedonia, artificial lake of Šum spring	41.18277°	20.63196°	5417	UGSB 0292	EU818821	JN794307
MK03/1	*Radix labiata* (Rossmässler, 1835)	Macedonia, Lubanište, creek flowing into Lake Ohrid	40.91453°	20.75881°	5340	UGSB 0282	EU818810	JN794308
MM01/1	*Radix* sp.	Myanmar, Kachin, Lake Indawgyi	25.12667°	96.29566°	11138	UGSB 7496		JN794309
MM02/1	*Radix* sp.	Myanmar, Kachin, Lake Indawgyi	25.13807°	96.30114°	11139	UGSB 7497		JN794310

MM03/1	*Radix* sp.	Myanmar, Kachin, Lake Indawgyi	25.20664°	96.32714°	11140	UGSB 7498	JN794493	JN794311
MM04/1	*Radix* sp.	Myanmar, Shan, Lake Inle	20.59090°	96.88020°	11141	UGSB 7499	JN794494	JN794312
MM05/1	*Radix* sp.	Myanmar, Shan, Lake Inle	20.52425°	96.89895°	11142	UGSB 7500		JN794313
MM06/1	*Radix* sp.	Myanmar, Shan, Lake Inle	20.56552°	96.90389°	11143	UGSB 7501		JN794314
MM06/2	*Radix* sp.	Myanmar, Shan, Lake Inle	20.56552°	96.90389°	11144	UGSB 7502		JN794315
MM07/1	*Radix* sp.	Myanmar, Shan, Lake Inle	20.61049°	96.90382°	11145	UGSB 7503		JN794316
MW01/1	*Radix natalensis* (Krauss, 1848)	Malawi, Karonga (BOMA), dam lake at Lake Malawi	-9.97632°	33.93944°	6120	UGSB 0304	EU818835	JN794317
NP01/1	*Radix* sp.	Nepal, Bheri Zone, Banke District, tributary of Rapti River	28.06667°	81.71667°	11233	UGSB 7504		JN794318
NP02/1	*Radix* sp.	Nepal, Bagmati Zone, Lalitpur District, Godawari, basin in botanic garden	27.60000°	85.40000°	11238	UGSB 7505		JN794319
NP02/2	*Radix* sp.	Nepal, Bagmati Zone, Lalitpur District, Godawari, basin in botanic garden	27.60000°	85.40000°	12654	UGSB 7506		JN794320
NP03/1	*Radix* sp.	Nepal, Bagmati Zone, Lalitpur District, Taudaha Lake	27.65000°	85.15000°	11239	UGSB 7507	JN794495	JN794321
NP04/1	*Radix* sp.	Nepal, Bheri Zone, Banke District, Nepalgunj, pond near road	28.04333°	81.60972°	11240	UGSB 7508	JN794496	JN794322
NP05/1	*Radix* sp.	Nepal, Bagmati Zone, Lalitpur District, rice fields	27.48488°	85.27740°	11241	UGSB 7509	JN794497	JN794323
NP06/1	*Radix* sp.	Nepal, Mechi Zone, Taplejung District, rice fields	27.45300°	87.70633°	11242	UGSB 7510	JN794498	JN794324
NP06/2	*Radix* sp.	Nepal, Mechi Zone, Taplejung District, rice fields	27.45300°	87.70633°	12655	UGSB 7511	JN794499	JN794325
NP07/1	*Radix* sp.	Nepal, Koshi Zone, Morang District, small river near Urlabari	26.66361°	87.61556°	11243	UGSB 7512	JN794500	JN794326
NP07/2	*Radix* sp.	Nepal, Koshi Zone, Morang District, small river near Urlabari	26.66361°	87.61556°	12656	UGSB 7513	JN794501	JN794327
NP08/1	*Radix* sp.	Nepal, Mahakali Zone, Kanchanpur District, Chandara River	28.94778°	80.26056°	11244	UGSB 7514	JN794502	JN794328
NP09/1	*Radix* sp.	Nepal, Mahakali Zone, Kanchanpur District, Chandara River	28.94778°	80.26056°	11245	UGSB 7515	JN794503	JN794329
NP10/1	*Radix* sp.	Nepal, Rapti Zone, Dang Deukhuri District, river	27.90306°	82.34417°	11247	UGSB 7516		JN794330
NP10/2	*Radix* sp.	Nepal, Rapti Zone, Dang Deukhuri District, river	27.90306°	82.34417°	12658	UGSB 7517		JN794331
NP11/1	*Radix* sp.	Nepal, Karnali Zone, Mugu District, Lake Rara	29.54207°	82.20625°	11249	UGSB 7518		JN794332
NP11/2	*Radix* sp.	Nepal, Karnali Zone, Mugu District, Lake Rara	29.54207°	82.20625°	12659	UGSB 7519		JN794333
NP12/1	*Radix* sp.	Nepal, Seti Zone, Bajhang District, ponds	29.47972°	81.12778°	12660	UGSB 7520	JN794504	JN794334
NP12/2	*Radix* sp.	Nepal, Seti Zone, Bajhang District, ponds	29.47972°	81.12778°	12661	UGSB 7521	JN794505	JN794335
NP13/1	*Radix* sp.	Nepal, Narayani Zone, Chitwan District, wetland	27.61361°	84.42222°	12662	UGSB 7522	JN794506	JN794336
NP14/1	*Radix* sp.	Nepal, Bheri Zone, Banke District, Dunai River	28.06639°	81.70389°	12663	UGSB 7523	JN794507	JN794337
RU01/1	*Radix auricularia* (Linnaeus, 1758)	Russia, Krasnodar Krai, channel near Golubitskaya	45.29897°	37.29839°	11106	UGSB 7524	JN794508	JN794338
RU02/1	*Radix auricularia* (Linnaeus, 1758)	Russia, Irkutsk Oblast, Irkutsk Dam	52.24257°	104.33123°	11299	UGSB 7525		JN794339
RU02/2	*Radix auricularia* (Linnaeus, 1758)	Russia, Irkutsk Oblast, Irkutsk Dam	52.24257°	104.33123°	11300	UGSB 7526		JN794340
RU03/1	*Radix auricularia* (Linnaeus, 1758)	Russia, Omsk Oblast, Omsk, waterbody near television factory	54.91667°	73.36667°	10949	UGSB 7527	JN794509	JN794341
RU04/1	*Radix zazurnensis* (Mozley, 1934)	Russia, Altai Republic, Lake Teletskoye	51.76667°	87.25000°	10953	UGSB 7528	JN794510	JN794342
RU05/1	*Radix auricularia* (Linnaeus, 1758)	Russia, Omsk Oblast, Omsk, Cheredovoye Lake	54.91667°	73.36667°	10956	UGSB 7529	JN794511	JN794343
TH01/1	*Radix* sp.	Thailand, Loei Province, ditch east of Chiang Khan	17.88830°	101.91300°	9379	UGSB 7530	JN794512	JN794344
TH01/2	*Radix* sp.	Thailand, Loei Province, ditch east of Chiang Khan	17.88830°	101.91300°	9381	UGSB 7531		JN794345
TJ01/1	*Radix auricularia* (Linnaeus, 1758)	Tajikistan, Gorno-Badakhshan, Lake Karakul	39.02164°	73.48873°	11552	UGSB 7532	JN794513	JN794346
TR01/1	*Radix* sp. 3 sensu Albrecht et al. [45]	Turkey, Isparta, Aglasun, water channel	37.64733°	30.53066°	4351	UGSB 0312	EU818802	JN794347
VN01/1	*Radix* sp.	Vietnam, Kon Tum Province, rice fields south of Dak Glei	14.98970°	107.75500°	9370	UGSB 7533	JN794514	JN794348
VN01/2	*Radix* sp.	Vietnam, Kon Tum Province, rice fields south of Dak Glei	14.98970°	107.75500°	9371	UGSB 7534		JN794349
VN01/3	*Radix* sp.	Vietnam, Kon Tum Province, rice fields south of Dak Glei	14.98970°	107.75500°	9372	UGSB 7535		JN794350
